# From Agriculture to Clinics: Unlocking the Potential of Magnetized Water for Planetary and Human Health

**DOI:** 10.7759/cureus.64104

**Published:** 2024-07-08

**Authors:** Piercarlo Minoretti, Enzo Emanuele

**Affiliations:** 1 Occupational Health, Studio Minoretti, Oggiono, ITA; 2 Clinical Pathology, 2E Science, Robbio, ITA

**Keywords:** autophagy, dermatology, dentistry, agriculture, hydrogen bonds, magnetized water

## Abstract

Magnetized water (MW) is a form of liquid water that has been exposed to a magnetic field to alter its hydrogen bonding structure, resulting in the formation of water molecule clusters of various sizes and configurations connected by hydrogen bonds. This magnetization process induces several changes in the physicochemical properties of water, such as increased pH, electrical conductivity, and dissolved oxygen content, as well as decreased surface tension, density, and evaporation temperature compared to untreated water. In this narrative review, we explore the effective utilization of MW in agriculture, where it has a well-established history of applications, and its potential for direct applications in the medical field, which are currently at the forefront of research.

MW is one of the most promising innovations for facilitating the transition from unsustainable to sustainable agriculture, which is expected to yield positive human health outcomes by promoting the consumption of less processed foods and reducing resource consumption. In addition to these indirect effects on human health, preclinical research utilizing animal models has demonstrated that water magnetization exerts beneficial effects on diabetes, renal function, bone health, and fertility. These health benefits appear to stem from the ability of MW to increase the activity of antioxidant enzymes while decreasing lipid peroxidation and inflammatory markers. In terms of direct human applications, MW has been primarily studied in the fields of dentistry and dermatology. MW mouthrinse has consistently shown efficacy against *Streptococcus mutans*, with studies reporting comparable effects to chlorhexidine. In dermatology, the topical application of MW has demonstrated improvements in skin biophysical parameters, increased hair count and hair mass index, and promoted the healing of challenging wounds. Intriguingly, these effects on human skin seem to be mediated by local activation of autophagy, potentially through mild alkaline stress.

In conclusion, this review underscores the promising role of MW in promoting a holistic approach to planetary and human health. Future studies should focus on standardizing the magnetization process, exploring the molecular mechanisms underlying MW-induced autophagy, and investigating the potential of MW as a complementary strategy for treating human diseases characterized by impaired autophagy.

## Introduction and background

**Figure 1:**
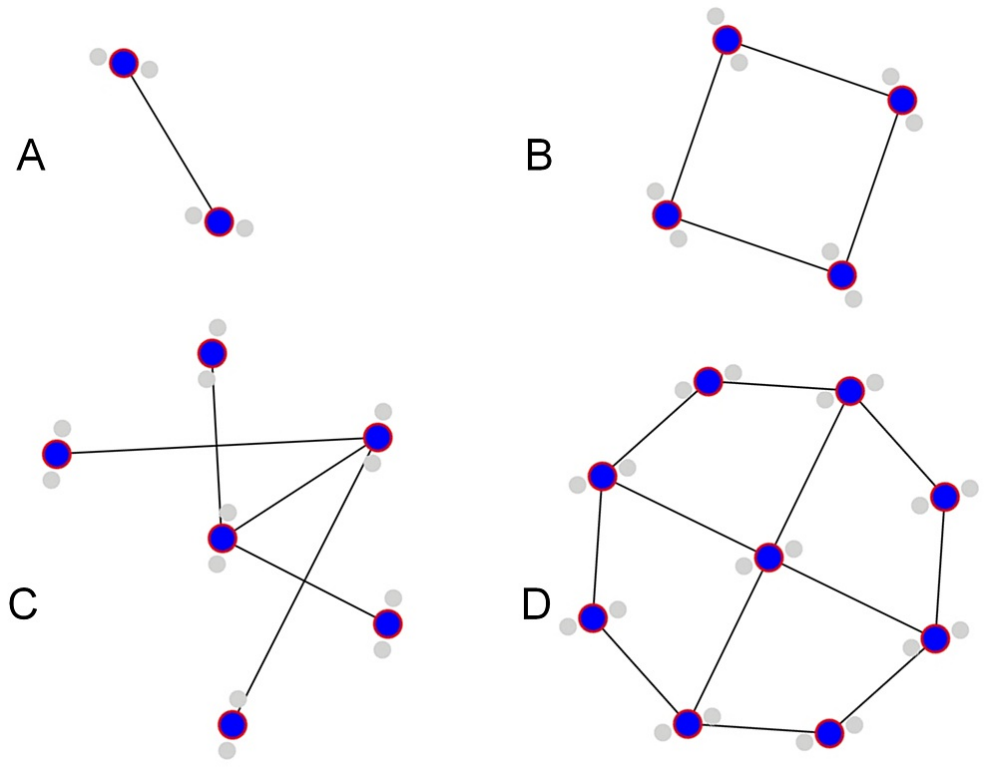
Exposure to a magnetic field can cause individual water molecules to form clusters with diverse sizes and configurations (A) Linear chain, (B) 4-cyclic structure, (C) bag-like arrangements, and (D) cyclic-boat assembly Image credits: Piercarlo Minoretti

Magnetized water (MW) is a form of liquid water that has undergone magnetic field treatment to alter its hydrogen bonding structure [1,2]. The production of MW typically involves the passage of water through a magnetic field generated by either permanent magnets or electromagnets. The apparatus employed for this purpose ranges from simple magnetic attachments affixed to pipes to more sophisticated electromagnetic water treatment systems [1,2]. The duration of magnetic exposure is a critical parameter influencing the resultant properties of MW. Exposure periods can vary significantly, spanning from seconds to several hours, contingent upon factors such as flow rate and magnetic field intensity. The magnitude of the applied magnetic field, which typically ranges from 0.1 to 1 Tesla, plays a pivotal role in determining the extent of water property modifications [1,2]. This magnetization process induces the formation of water molecule clusters with diverse sizes and configurations, interconnected via hydrogen bonds (Figure 1) [2,3]. These clusters exhibit a wide spectrum of complexity, ranging from small assemblages comprising a few molecules to larger aggregates consisting of hundreds of molecules [1,2]. The resulting molecular arrangements in MW differ significantly from both the structure of untreated liquid water and the highly ordered, hexameric configuration observed in ice [2].

The precise mechanisms underlying the formation of water clusters following magnetic field exposure are not fully elucidated, but they are believed to involve alterations in the strength of hydrogen bonds between individual water molecules [3]. Upon exposure to a magnetic field, water exhibits weakened intercluster bonds and strengthened intracluster bonds, resulting in a modest increase in overall hydrogen bonding [2,3]. This restructuring is evidenced by changes in the absorption of visible, ultraviolet, infrared, and Raman spectra of MW compared to untreated tap water [2]. Although the effects of magnetization on water are typically transient, with the structured state reverting to that of untreated water within minutes to hours after removal from the magnetic field, some highly stable forms of MW have been developed that maintain their structured state for several months [2,4]. For instance, an MW product treated with magnetic and light energy, along with selected minerals, has demonstrated long-term stability and has been evaluated in various biological systems [2].

The formation of clusters in water due to magnetization leads to several changes in its physicochemical properties. Studies have reported increases in pH, electrical conductivity, and dissolved oxygen content, as well as decreases in surface tension, density, and evaporation temperature in MW compared to untreated water [2,3,5]. Notably, the specific changes appear to be dependent on the strength of the magnetic field employed (500-1,000 G) [2]. These distinctive properties of MW have been linked to various biological effects that have been extensively explored in agricultural research and, more recently, in pilot medical applications. Currently, MW stands out as one of the most promising innovations for facilitating the transition from unsustainable to sustainable agriculture [1]. This transition is expected to yield positive human health outcomes by promoting the consumption of less processed foods and reducing resource consumption [6,7]. Evidence also suggests that many health-improving interventions originate in the soil [8]. For instance, sustainable agriculture can produce more diverse and phytochemically rich diets, leading to functional foods that may positively impact human health through various mechanisms, including gut microbiota modulation and reduced intestinal permeability [9]. In addition to these indirect effects on human health, preclinical and clinical research has recently started exploring the potential medicinal applications of MW.

In this narrative review, we will explore how MW can be effectively utilized in agriculture, where it has a well-established history of applications, and its potential for direct applications in the medical field, which are currently at the forefront of research. By examining the pathways through which MW technology can benefit both agriculture and medicine, we aim to highlight its promising role in promoting a holistic approach to a healthier planet and human population.

## Review

Search strategy

To compile relevant literature for this narrative review, a comprehensive search was conducted using the PubMed database, focusing on peer-reviewed articles. The search was inclusive, with no restrictions on language or publication date. The final update to the literature search was performed on June 30, 2024, ensuring the inclusion of the most recent applicable research. The search strategy utilized a combination of key terms related to the effects of MW on two fields of interest: agriculture and medicine. These terms were paired with "magnetized water" to ensure the results were specific to this technology. The search query was formulated as follows: (magnetized water [Title/Abstract] OR magnetic water treatment [Title/Abstract] OR magnetic fields [Title/Abstract]) AND (agriculture [Title/Abstract] OR crops [Title/Abstract] OR plants [Title/Abstract] OR health [Title/Abstract] OR medicine [Title/Abstract] OR disease [Title/Abstract]). The results were then carefully screened for relevance to the specific topics addressed in this review. Following this initial screening process, the reference lists of the selected articles were manually examined to identify any additional relevant studies that may have been missed in the primary search. Ultimately, the final reference list comprised 61 articles that were deemed most pertinent to the topics covered in this review. These articles formed the foundation of the evidence presented and discussed throughout the review.

Magnetized water in agriculture: a sustainable strategy to improve crop quality and yield

**Figure 2:**
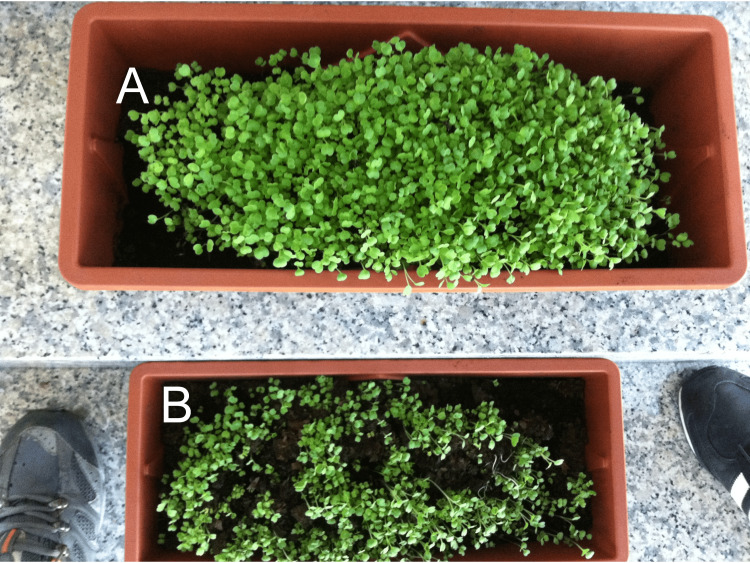
Comparison of domestic arugula (Eruca vesicaria) growth under different irrigation treatments (A) Plants irrigated with magnetized water exhibit enhanced growth and vigor compared to (B) plants irrigated with untreated water. Both panels depict plants at the same growth stage and under identical environmental conditions. Image credits: Enzo Emanuele

MW is gaining increasing recognition as an eco-friendly and sustainable technology for enhancing plant growth and agricultural productivity [1]. Numerous studies have demonstrated the positive effects of irrigating crops with water that has been passed through a magnetic field [10,11]. For instance, the utilization of magnetically treated brackish water for wheat irrigation resulted in substantial enhancements, specifically a 27% increase in biological yield, a 34% increase in straw yield, and a 19% increase in grain yield, relative to irrigation with untreated water [12]. In addition, MW has also been found to significantly improve the growth and yield of various other crops, including lentils [13], durum wheat [13], barley [14], rice [15], mustard [16], bean [17], lettuce [18], and cotton [19], among others. Notably, plants irrigated with MW consistently exhibit increased plant height, leaf area, root length, and overall biomass production compared to those irrigated with non-magnetized water [1]. Figure 2 illustrates the striking difference in growth between domestic arugula (*Eruca vesicaria*) plants irrigated with MW and those receiving untreated water.

Several studies have attempted to identify the key mechanisms by which MW improves plant growth. One such process is the enhancement of photosynthetic activity. MW has been found to increase the content of chloroplast pigments such as carotenoids, chlorophyll a, and chlorophyll b in plant leaves, leading to higher photosynthetic rates and better overall plant health [1,20]. For instance, tobacco plants irrigated with MW showed a significant increase in photosynthetic rate and protein content compared to those irrigated with non-MW [21]. Furthermore, the use of MW has been found to activate enzymes and hormones involved in the growth process [22,23]. This leads to improved seedling emergence and early vegetative development. In an experiment on maize growth, the emergence rate index increased from 7.6 to 10.2, 9.1 to 11.1, 10.3 to 13.3, and 11.8 to 13.3 when applying magnetized water at four different salinity levels [24]. MW has also been shown to improve the mobility and uptake of essential plant nutrients such as phosphorus, potassium, and zinc, resulting in better nutrient content in plants and enhanced growth criteria [24]. Further research has revealed that irrigation with magnetically treated water can beneficially modify soil properties. Notably, irrigation with MW has been observed to enhance soil electrical conductivity while simultaneously reducing soil pH [25]. Additionally, MTW application facilitates the leaching of excess soluble salts from the soil profile and improves soil moisture retention capacity [25]. These changes in soil properties create a more favorable environment for plant growth. Moreover, MW has shown potential in alleviating abiotic stresses such as salinity and drought in crops [15]. Another study demonstrated that magnetic treatment of irrigation water helped reduce salt stress in tomato plants grown under saline conditions [26]. MW also enhances the activity of antioxidant enzymes and reduces oxidative damage in mung beans under stress conditions [27]. At the cellular level, MW maintains the integrity of chloroplasts and thylakoid membranes compared to non-magnetized water [21]. This contributes to the overall improvement in plant health and growth observed in plants irrigated with MW.

**Figure 3:**
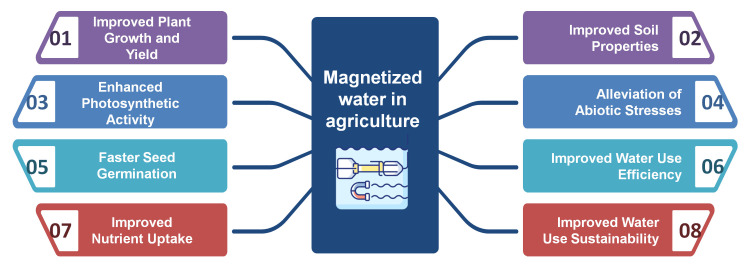
Schematic representation of the effects of magnetized water on plants Magnetized water enhances seed germination, root development, plant growth, and crop yield through improved nutrient uptake and photosynthetic efficiency. Additionally, magnetized water reduces the adverse effects of abiotic stresses such as salinity and drought, leading to enhanced plant tolerance and resilience under challenging environmental conditions. Image credits: Enzo Emanuele

One of the most important properties of MW for agricultural applications is its sustainability, which can be ascribed to three key factors. First, the use of MW enhances water use efficiency by improving the water absorption capacity of soil and plants, allowing for water savings [11]. This is particularly beneficial in arid and semiarid regions facing water scarcity. By optimizing water usage, MW can help conserve this precious resource and reduce the environmental impact of agriculture [1]. Second, magnetic treatment of low-quality water sources, such as brackish, saline, or contaminated water, can serve as an innovative approach to render previously unsuitable water viable for agricultural use [28]. This technique has the potential to significantly expand the available water supply for irrigation, thereby reducing the pressure on increasingly scarce freshwater resources. Finally, by boosting crop productivity, MW can reduce the need for chemical fertilizers and pesticides, thus minimizing environmental impact [1,29]. The enhanced nutrient uptake and improved plant health associated with MW irrigation can lessen the reliance on agrochemicals, promoting a more sustainable and eco-friendly approach to agriculture. Based on the reviewed evidence, the use of MW in agriculture can be considered an eco-friendly technology that enhances water use efficiency and sustainability while simultaneously improving crop yield, making it a promising solution to the challenges posed by climate change and water scarcity (Figure 3).

Application in the medical field: preclinical and clinical studies

While the beneficial physicochemical modifications induced in water by magnetization have been extensively leveraged in agriculture, their application in the medical field has remained limited. However, there has been a recent increase in interest in this area. In rodent models, the systemic administration of MW has been investigated for its effects on diabetes mellitus, renal dysfunction, bone health, fertility, and liver parameters. Lee and Kang [30] investigated the effects of MW supplementation on blood glucose, DNA damage, antioxidant status, and lipid profiles in rats with streptozotocin (STZ)-induced diabetes. The rats were divided into three groups: a control group without diabetes, a diabetes group with STZ-induced diabetes, and an MW group that received magnetized water for eight weeks after diabetes induction. The MW group showed decreased blood glucose levels from the fourth week onward compared to the diabetes group, as well as significantly reduced glycated hemoglobin levels [30]. MW supplementation also significantly decreased blood and liver DNA damage in diabetic rats, although it did not affect plasma insulin levels, antioxidant enzyme activities, or lipid profiles [30].

Zayed et al. [31] examined *Ginkgo biloba* (GB) and MW in relation to their protective effects against nephrotoxicity associated with experimental type 2 diabetes in rats [31]. Diabetes was induced by feeding rats a high-fat diet for four weeks, followed by STZ injection, and the rats were divided into four groups: control, diabetic, diabetic + GB, and diabetic + MW. Both GB and MW treatment significantly reduced urea, creatinine, and glucose levels, attenuated glomerular and tubular injury, and normalized glutathione reductase and superoxide dismutase 2 contents. The authors concluded that GB and MW protected type 2 diabetic rat kidneys from nephrotoxic damage by reducing hyperlipidemia, uremia, and oxidative stress [31].

In a separate study, Saleh et al. [32] investigated the effects of GB extract and MW on pancreatic β-cells in type 2 diabetic rats. Results showed that both treatments, particularly GB, increased β-cell mass and insulin expression while also reducing the expression of antioxidant enzymes in pancreatic tissue [32].

Another investigation by Balieiro Neto et al. [33] examined the effects of water treated with a magnetic field on bone mineral density (BMD), bone mineral content (BMC), bone area (BA), bone resistance (BR), blood gas analysis, blood viscosity, and blood biochemical profile in rats. Forty-eight Wistar rats were divided into control and MW-treated groups, each further subdivided into three groups to evaluate different consumption periods (15, 30, and 45 days). While no significant differences were found in water intake, dry matter intake, BA, or femoral head resistance between the groups, the MW-treated group showed a higher anion gap in arterial blood. A significant interaction was observed between the MW consumption period and BR, BMD, and BMC, with increases in BR (midshaft), BMD, and BMC after 30 and 45 days of consumption, suggesting that consuming MW for 45 days may effectively improve BMD, BMC, and BR in rats [33].

Hafizi et al. [34] investigated the effects of MW on the height of epithelial cells in the pre-implantation stage endometrium and fallopian tube, as well as the number of corpus lutea in female mice. Eighty female mice were divided into two groups: a control group that drank normal water and an experimental group that drank MW for two weeks. The results showed a significant increase in the mean number of corpus lutea and the height of epithelial cells in the fallopian tube in the MW group compared to the control group, while the increase in uterus epithelial cell height was not significant [34].

Recently, Elmoslemany et al. [35] examined the effects of MW and microwave-treated water on rats' liver tissues. Rats administered microwave-treated water showed significant increases in liver function enzymes, bilirubin levels, and oxidative stress parameters, as well as notable histopathological changes in liver tissues. In addition, microwave-treated water also induced epigenetic effects, with significant changes in the expression of various genes, including the downregulation of glutathione-S-transferase, cytochrome P450, and metallothionein, as well as the upregulation of amylase and HDAC3. In contrast, rats administered MW showed no clear changes in their liver tissues compared to tap water, suggesting that MW did not induce the stress that was induced in the presence of microwave-treated water [35].

Table 1 summarizes the published studies investigating the effects of MW on various animal models.

**Table 1:** Summary of preclinical studies on the effects of magnetized water in animal models Abbreviations: STZ, streptozotocin; MW, magnetized water; GB, *Ginkgo biloba*; BA, bone area; BR, bone resistance; BMD, bone mineral density; BMC, bone mineral content

Authors and year of publication	Animal model	Experimental groups	Study duration	Key findings
Lee and Kang (2013) [30]	Rats with STZ-induced diabetes	Control, diabetes, diabetes + MW	8 weeks	Decreased blood glucose levels from week 4 in MW group; reduced glycated hemoglobin levels; decreased blood and liver DNA damage; no effect on plasma insulin, antioxidant enzyme activities, or lipid profiles
Zayed et al. (2018) [31]	Rats with high-fat diet and STZ-induced diabetes	Control, diabetes, diabetes + GB, diabetes + MW	4 weeks	Reduced levels of urea, creatinine, and glucose in GB- and MW-treated groups; attenuated glomerular and tubular injury in GB- and MW-treated groups
Saleh et al. (2019) [32]	Rats with high-fat diet and STZ-induced diabetes	Control, diabetes, diabetes + GB, diabetes + MW	4 weeks	Increased β-cell mass and insulin expression in GB- and MW-treated groups, improved pancreatic antioxidant status in GB- and MW-treated groups
Balieiro Neto et al. (2017) [33]	Wistar rats	Control, MW	Three consumption periods (15, 30, 45 days)	No significant differences in water intake, dry matter intake, BA, or femoral head resistance; higher anion gap in arterial blood in the MW group; increased BR (midshaft), BMD, and BMC after 30 and 45 days of MW consumption
Hafizi et al. (2014) [34]	Female mice	Control, MW	2 weeks	Increased mean number of corpus lutea and height of epithelial cells in the fallopian tube in the MW group, insignificant increase in uterus epithelial cell height
Elmoslemany et al. (2023) [35]	Rats	Tap water, MW, microwave-treated water	2 months	Microwave-treated water: increased liver functioning enzymes, bilirubin levels, oxidative stress parameters, histopathological changes, and epigenetic effects; MW: no clear changes in liver tissues compared to tap water

Application in the medical field: oral health

The potential effects of MW on human health have been a topic of scientific inquiry since the 1980s. In a seminal study conducted in China, Wu [36] analyzed 114 pediatric cases and reported the potential therapeutic applications of MW in the treatment of ascariasis. However, following this initial observation, the majority of clinical studies on MW have primarily focused on its topical effects, particularly in the fields of oral health and dermatology.

In the first published study in the field of dentistry, Johnson et al. [37] investigated the effects of a MW oral irrigator (Hydro Floss) on plaque accumulation, calculus formation, and gingival health in adult subjects. The results showed that irrigation with MW resulted in 64% less calculus buildup compared to the control group after three months of use (p<0.02). The reduction in gingival inflammation was 27%, although this difference did not reach statistical significance [37].

Gupta and Bhat [38] compared the antibacterial efficacy of 0.2% chlorhexidine mouthrinse, considered the gold standard, with MW on *Streptococcus mutans* in 50 children aged 5-12 years. The subjects were divided into four groups: chlorhexidine, two MW groups (24 hours magnetization) rinsing for one and three minutes, and a MW group (72 hours magnetization) rinsing for three minutes. Saliva samples were collected at baseline and after rinsing to assess *S. mutans *counts. The results showed that the 72-hour MW group had a reduction in *S. mutans *count almost on par with the chlorhexidine group [38].

In a similar study, Goyal et al. [39] assessed the antimicrobial effectiveness of MW as a mouthwash on *S. mutans *colony count. A sample of 30 children aged 7-12 years used 10 mL of 72-hour MW for three minutes twice daily for two weeks, with plaque and saliva samples collected at baseline and one-week and two-week intervals. The results showed a highly significant reduction in *S. mutans* count in both plaque and saliva after one and two weeks compared to baseline. The authors concluded that MW is an effective mouthwash against* S. mutans*, particularly in plaque, and can be used as an adjunct to commercially available mouthwashes [39].

In a double-blinded randomized control clinical study, Nagpal et al. [40] compared the effectiveness of MW and 0.2% chlorhexidine as a mouthrinse in children aged 12-15 years for plaque and gingivitis inhibition over three weeks. A total of 20 children were randomized into two groups, each comprising 10 children who were asked to rinse with their respective mouthwash. Plaque index (PI) scores and gingival index (GI) scores were evaluated at baseline, two weeks, and three weeks for each child. The results showed a statistically significant reduction in mean PI and GI scores for both MW (p=0.0001) and chlorhexidine groups (p=0.0001) at two and three weeks, with no adverse effects [40].

In a recent clinical trial, Nezam et al. [41] compared the effects of MW and 0.2% chlorhexidine mouthwash on gingivitis and plaque prevention in children aged 12-15 years over a period of 21 days. A total of 24 children were randomly divided into two groups, with one group using MW as a mouthrinse and the other using 0.2% chlorhexidine. The PI and GI were analyzed at baseline, 14 days, and 21 days, and adverse effects such as bitter taste and brownish discoloration were recorded. The results showed that both MW and chlorhexidine were similarly effective in managing periodontal and gingival infections; however, MW had fewer side effects, making it a safer and more acceptable alternative to chlorhexidine mouthwashes, especially in children [41].

Table 2 summarizes the clinical studies investigating the effects of MW on oral health.

**Table 2:** Summary of clinical studies investigating the effects of magnetized water on oral health Abbreviation: MW, magnetized water

Authors and year of publication	Subjects	Groups	Duration	Outcome measures	Results
Johnson et al. (1998) [37]	Adults	MW oral irrigator versus control	3 months	Plaque, calculus, gingival health	64% less calculus in the MW group (p<0.02), 27% reduction in gingival inflammation in the MW group (not significant)
Gupta and Bhat (2011) [38]	Children aged 5-12 years	0.2% chlorhexidine versus MW (24 hours) 1 minute versus MW (24 hours) 3 minutes versus MW (72 hours) 3 minutes	Single use	*S. mutans* count in saliva	The MW (72 hours) group had a reduction in *S. mutans* almost on par with chlorhexidine
Goyal et al. (2017) [39]	Children aged 7-12 years	MW (72 hours) 3 minutes, twice daily	2 weeks	*S. mutans* count in plaque and saliva	Highly significant reduction in *S. mutans* in plaque and saliva at 1 and 2 weeks versus baseline
Nagpal et al. (2020) [40]	Children aged 12-15 years	MW versus 0.2% chlorhexidine	3 weeks	Plaque index, gingival index	Significant reduction in plaque index and gingival index at 2 and 3 weeks for both MW (p=0.0001) and chlorhexidine (p=0.0001)
Nezam et al. (2022) [41]	Children aged 12-15 years	MW versus 0.2% chlorhexidine	21 days	Plaque index, gingival index, side effects	MW and chlorhexidine similarly effective for plaque index and gingival index, MW had fewer side effects

Application in the medical field: dermatology

In the field of dermatology, MW delivered in topical formulations has recently shown promise as a novel strategy for improving the biophysical properties of the skin and promoting hair growth, likely mediated through autophagy activation. In an open-label study, Minoretti et al. [42] investigated the effects of topically applying a serum containing saline MW on facial and neck skin appearance in 20 females. The MW serum significantly improved skin hydration, reduced transepidermal water loss, decreased sebum content, and lowered melanin and erythema indices after 12 weeks. A molecular substudy on 10 participants revealed that the serum increased beclin-1 levels by 38% and decreased mammalian target of rapamycin (mTOR) levels by 24% in skin biopsies, indicating the activation of cutaneous autophagy. Collectively, these findings indicated that MW applied topically in a serum formulation shows promise in enhancing skin biophysical parameters for females seeking to improve their facial and neck skin appearance [42].

In a separate study, García Martín et al. [43] investigated the effects of topically applied saline MW on androgenetic alopecia in 20 Caucasian males. Over 12 weeks, participants applied a lotion containing 95% saline MW daily, resulting in significant increases in hair count and hair mass index. Molecular analysis of scalp biopsies from a subgroup of 10 males showed increased levels of autophagy markers beclin-1 and microtubule-associated protein 1A/1B-light chain 3 (LC3B). The authors concluded that saline MW effectively activated scalp autophagy and improved hair growth in males with mild to moderate androgenetic alopecia [43].

Finally, a recent case series documented the successful management of five non-infected, difficult-to-heal wounds in elderly patients using a topical autophagy-stimulating gel containing 95% saline MW [44]. The treated wounds, which included pressure ulcers, venous ulcers, and trauma-related injuries, had shown minimal or no improvement with standard wound therapies over a prolonged period. Application of the autophagy-stimulating gel promoted wound healing, as indicated by reduced fibrous and necrotic tissue, granulation tissue formation, re-epithelialization, and partial or complete wound closure. These preliminary case studies suggest that a topical gel containing saline MW, which promotes autophagy, may aid the healing of chronic wounds in elderly patients and warrant further investigation as a potential addition to existing wound care treatments for the aging population [44].

Table 3 provides a summary of the clinical studies investigating the effects of MW in the field of dermatology.

**Table 3:** Summary of clinical studies investigating the effects of magnetized water in the field of dermatology Abbreviations: MW, magnetized water; TEWL, transepidermal water loss; mTOR, mammalian target of rapamycin; LC3B, microtubule-associated protein 1A/1B-light chain 3

Authors and year of publication	Subjects	Groups	Duration	Outcome measures	Results
Minoretti et al. (2023) [42]	Adult females	Single group using serum with saline MW	12 weeks	Skin hydration, TEWL, sebum content, melanin and erythema indices; beclin-1 and mTOR levels in skin biopsies	Improved skin hydration, reduced TEWL, decreased sebum, melanin, and erythema; 38% increase in beclin-1 and 24% decrease in mTOR, indicating activated cutaneous autophagy
García Martín et al. (2023) [43]	20 Caucasian males with androgenetic alopecia	Single group using lotion with 95% saline MW	12 weeks	Hair count, hair mass index; beclin-1 and LC3B levels in skin biopsies	Increased hair count and hair mass index; increased beclin-1 and LC3B, indicating enhanced scalp autophagy
Minoretti et al. (2024) [44]	Five elderly patients with non-infected, difficult-to-heal wounds	Case series using topical gel with 95% saline MW	The gel was applied regularly until the wound had fully healed or showed significant clinical improvement	Wound healing parameters (fibrous and necrotic tissue, granulation, re-epithelialization, wound closure)	Promoted wound healing; reduced fibrous and necrotic tissue; increased granulation, re-epithelialization, and partial or complete wound closure

Discussion

MW has emerged as a promising eco-friendly approach to address significant challenges across various domains. To date, the majority of research has focused on its application in sustainable agriculture. As highlighted in a recent review by Dobránszki et al. [1], utilizing MW for irrigation has demonstrated the ability to enhance plant growth and development while simultaneously mitigating abiotic stressors, such as water scarcity and high salinity levels. Notably, MW enables water savings surpassing 10% in agricultural irrigation systems while concurrently increasing crop yield and improving produce quality [1]. Consequently, the potential of water magnetization is being investigated as a feasible solution to address the urgent issue of freshwater shortages [10,11]. Furthermore, MW can be seamlessly incorporated into phytoremediation techniques [45], which aim to effectively decontaminate polluted soil and water resources. The integration of MW in these processes has the potential to enhance the efficiency and effectiveness of phytoremediation efforts, contributing to the restoration of contaminated ecosystems [45]. Collectively, the far-reaching impacts of MW contribute significantly to mitigating the water scarcity challenges that are exacerbated by the ongoing climate change crisis [1]. By promoting sustainable agriculture practices, reducing freshwater consumption, and facilitating the remediation of polluted resources, MW presents a promising approach to address some of the most pressing environmental issues of our time.

Agriculture is also intricately intertwined with health; unfortunately, the connections between these two crucial sectors are frequently overlooked, as Hawkes and Ruel [46] observed nearly 20 years ago. Primarily, agriculture plays a pivotal role in promoting human health by producing the world's food, fiber, and materials for shelter [46]. In addition, the agricultural sector can also be associated with various medical challenges, including malnutrition, malaria, foodborne illnesses, infectious diseases, livestock-related ailments, occupational disorders, and non-communicable diseases [46,47]. Given the intricate relationship between agriculture and human health, and the positive impact of MW in agricultural applications, it becomes evident that the utilization of water magnetization in the agricultural field may indirectly yield favorable outcomes for human health. MW also has the potential to enhance crop growth and nutrient uptake [10-21], resulting in higher levels of essential vitamins, minerals, and antioxidants in agricultural produce. This, in turn, may contribute to a more nutritious food supply, which is paramount for the prevention of chronic diseases [48]. Furthermore, MW can facilitate the production of functional foods that are rich in bioactive compounds, which have the potential to reduce systemic inflammation and enhance immune function [49]. By improving crop growth and resilience [1], MW may also help mitigate the overuse of chemical pesticides, which can have detrimental effects on human health, including neurotoxicity and developmental toxicity.

**Figure 4:**
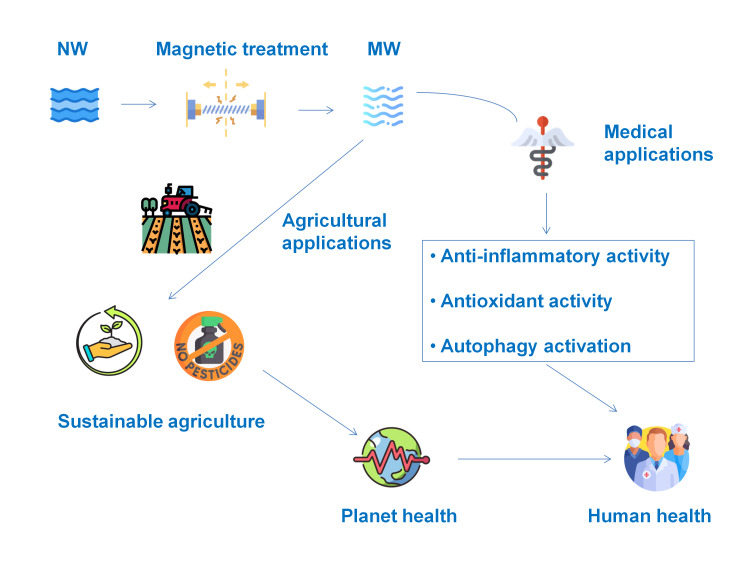
Schematic representation of the indirect and direct health benefits of magnetized water In agricultural applications, magnetized water can facilitate sustainable practices, potentially reducing pesticide usage, thus contributing to planetary health and indirectly benefiting human health. Moreover, magnetized water has proposed direct medical applications attributed to its anti-inflammatory, antioxidant, and autophagy-activating properties, ultimately leading to direct human health benefits. Abbreviations: NW, normal water; MW, magnetized water Image credits: Enzo Emanuele

Beyond its advantageous indirect effects on human health through agricultural use, MW is attracting increasing scientific interest and research focus for its prospective direct therapeutic applications in medicine (Figure 4).

Although animal studies have investigated the systemic impacts of MW ingestion [30-35], human studies have been restricted to topical use to date [37-44]. Preclinical research utilizing animal models has demonstrated that water magnetization exerts beneficial effects on diabetes [30-32], renal function [31], bone health [33], and fertility [34]. These health benefits appear to stem from the ability of MW to increase the activity of antioxidant enzymes (e.g., catalase, glutathione peroxidase, and superoxide dismutase) while decreasing lipid peroxidation and inflammatory markers [30,32]. Notably, a recent study demonstrated the safety of systemic MW administration in animals, with no significant adverse effects on liver function and morphology, in contrast to microwave-treated water [35]. In terms of direct human applications, MW has been primarily studied in the fields of dentistry [37-41] and dermatology [42-44]. MW mouthrinse has consistently shown efficacy against *S. mutans*, with studies reporting comparable effects to 0.2% chlorhexidine [38-41]. This antibacterial activity is likely mediated through alterations in the oral environment and/or direct bacterial inhibition. In this regard, MW may modify the pH and buffering capacity of saliva, creating conditions less conducive to *S. mutans* growth [39]. In addition, the physical and chemical changes induced by magnetization [2,3,5] could potentially increase intracellular leakage of proteins and DNA from bacterial cells.

In the field of dermatology, the topical application of MW has demonstrated improvements in skin biophysical parameters [42], increased hair count and hair mass index [43], and promoted the healing of challenging wounds [44]. Intriguingly, these effects on human skin seem to be mediated by local activation of autophagy, potentially through mild alkaline stress [42,43]. Autophagy, an evolutionarily conserved cellular process, plays a pivotal role in maintaining cellular homeostasis by degrading and recycling damaged or dysfunctional cellular components, such as proteins and organelles, through lysosomal digestion [50]. Dysfunction of autophagy is implicated in numerous age-related diseases [51]. As a drug-free approach to activating autophagy, MW holds promise as a complementary strategy in various human diseases where impaired autophagy plays a crucial pathogenic role. Autophagy also has a complex interplay with the immune system, with implications for infectious diseases, autoimmunity, and inflammation [52]. Activation of autophagy can help eliminate intracellular pathogens by targeting them for lysosomal degradation [53]. Furthermore, autophagy modulates the innate and adaptive immune responses by regulating cytokine production and antigen presentation [52]. Defects in autophagy have been associated with increased susceptibility to infections and the development of autoimmune disorders [50,52]. Another way in which autophagy promotes health is by removing misfolded proteins, a hallmark of several neurodegenerative diseases, including Alzheimer's disease and Parkinson's disease [54]. Moreover, autophagy plays a critical role in regulating cellular metabolism and energy homeostasis [55]. During periods of nutrient deprivation or cellular stress, autophagy is upregulated to recycle cellular components and generate energy-rich compounds, such as amino acids and fatty acids, which can be used for cell survival [56]. This process is particularly important in maintaining the function of metabolically active tissues, such as the liver, muscle, and adipose tissue [55,56]. Hence, dysregulation of autophagy has been implicated in metabolic disorders, such as obesity and diabetes [57].

Based on these observations, several promising future research directions can be highlighted. First, investigating the molecular mechanisms underlying MW-induced autophagy activation in human cells is a crucial area for further exploration. This may involve elucidating the specific signaling pathways and cellular processes that are modulated by MW, as well as identifying the key molecular players involved in these processes. Second, exploring the potential of MW as a complementary strategy for treating age-related diseases characterized by impaired autophagy, such as neurodegenerative diseases, autoimmune conditions, and metabolic disorders, is another promising avenue for future research. Third, investigating the potential of combining MW with other therapeutic approaches, such as pharmacological agents [58] or lifestyle aspects [59], to enhance its effects on autophagy activation is an important area for future studies. This may involve examining the synergistic or additive effects of MW when used in conjunction with other autophagy-modulating interventions, as well as assessing the safety and efficacy of such combinatorial approaches. Importantly, future studies should also attempt to standardize the technique used for magnetizing water and assess how changing the magnetization field may modify the biological effects of magnetized water. This will require a systematic evaluation of the various parameters involved in the magnetization process, such as the strength and duration of the magnetic field, as well as the type of water used and the storage conditions employed. By establishing standardized protocols and optimizing the magnetization process, researchers can ensure the reproducibility and reliability of their findings, paving the way for the development of effective MW-based interventions for promoting autophagy and improving human health.

Limitations

This manuscript exemplifies a narrative review, acknowledging inherent limitations and caveats associated with this approach [60]. While systematic reviews are generally regarded as superior in methodological rigor, narrative reviews maintain significant value within the medical community [60]. Indeed, prominent journals continue to publish a higher proportion of narrative reviews compared to their systematic counterparts [61]. It is important to note that an exhaustive analysis of MW's effects on agriculture fell beyond the purview of this study, as recent publications have already addressed this topic comprehensively [1,10]. Notwithstanding these constraints, our review represents a pioneering effort to elucidate the translational potential of magnetized water from agricultural applications to medical contexts, thereby delineating promising avenues for future scientific inquiry.

## Conclusions

MW represents a multifaceted technology with wide-ranging applications in agriculture and burgeoning therapeutic potential in the medical field. Its capacity to augment crop yield and quality while concomitantly promoting human health indirectly renders it an invaluable asset for sustainable agricultural practices. The direct utilization of MW in clinical settings, particularly as an autophagy activator, constitutes a promising avenue for future scientific inquiry. Nevertheless, rigorously controlled human clinical trials and the standardization of magnetization parameters are imperative to fully harness MW's potential as a safe and efficacious strategy within the realm of alternative and complementary medicine. By establishing a nexus between agriculture and medicine, MW presents a unique opportunity to foster a holistic approach to both planetary and human health.
